# Interactive weekly mobile phone text messaging plus motivational interviewing in promotion of breastfeeding among women living with HIV in South Africa: study protocol for a randomized controlled trial

**DOI:** 10.1186/s13063-017-2079-0

**Published:** 2017-07-17

**Authors:** Moleen Zunza, Mark F. Cotton, Lawrence Mbuagbaw, Richard Lester, Lehana Thabane

**Affiliations:** 1 Faculty of Medicine and Health Sciences, Department of Global Health, Centre for Evidence Based Health Care, Stellenbosch University, Francie Van Zyl Drive, PO Box 241, Cape Town, 8000 South Africa; 2 Research Institute, McGill University Health Centre, Montreal, QC Canada; 3 CIHR Canadian HIV Trials Network, 588-1081 Burrard Street, Vancouver, BC V6B 3E6 Canada; 4 Faculty of Medicine and Health Sciences, Department of Paediatrics and Child Health, Stellenbosch University, Francie Van Zyl Drive, PO Box 241, Cape Town, 8000 South Africa; 5 Department of Clinical Epidemiology and Biostatistics, McMaster University, Hamilton, ON Canada; 6 Biostatistics Unit, Father Sean O’Sullivan Research Centre, St Joseph’s Healthcare, Hamilton, ON Canada; 7 Global Health, Division of Infectious Diseases, Faculty of Medicine, University of British Columbia, Vancouver General Hospital, Vancouver, Canada

**Keywords:** HIV, infant feeding, mHealth, motivational interviewing, text messaging

## Abstract

**Background:**

South Africa recently phased out access to free formula milk in the public sector in support of breastfeeding for women living with HIV. Few women living with HIV in South Africa choose breastfeeding and among those who do, many stop breastfeeding early. We sought to explore the feasibility of using mobile phone text messaging coupled with motivational interviewing to enhance adherence to breastfeeding practices.

**Methods and design:**

A randomized, parallel group, single-center pilot trial. Electronic sequence generation and random allocation will be done centrally. Women of low socioeconomic status, from Cape Town, South Africa will be randomly assigned within 24 h of giving birth at a primary healthcare clinic to a structured weekly text message plus motivational interviewing and usual standard of care, using a permutation of different block sizes. Criteria for feasibility success will include: five participants recruited per week (over 12 weeks), about 75% of all eligible participants consent for study participation, complete evaluation of outcomes in at least 70% of all recruited participants, breastfeeding adherence rates of at least 70% in the intervention group, six months after delivery. Participants will be evaluated soon after giving birth and post-delivery at weeks 2, 6, 10, and 24. Primary analysis will follow the “intention-to-treat” principle. Sub-group analysis will be used to assess sub-group effects.

**Discussion:**

This pilot trial will evaluate the feasibility of conducting a larger trial on communication and support approaches to improve adherence to breastfeeding by HIV-infected women. Text messaging and motivational interviewing are simple interventions which may allow participants to access personalized adherence advice and support.

**Trial registration:**

ClinicalTrials.gov: NCT02949713. Registered on 26 October 2016; Pan African Clinical Trial Registry PACTR201611001855404. Registered on 8 November 2016.

**Electronic supplementary material:**

The online version of this article (doi:10.1186/s13063-017-2079-0) contains supplementary material, which is available to authorized users.

## Background

Since the detection of HIV transmission through breastfeeding, developed countries recommend formula feeding for HIV-infected women [1]. In resource-limited settings, where child mortality is mainly due to diarrhea, pneumonia, and malnutrition, the effectiveness of antiretroviral treatment (ART) in reducing the risk of HIV transmission through breastfeeding has largely resolved the dilemma on how HIV-infected women should feed their infants [2–4]. In such settings, the World Health Organization (WHO) recommends at least 12 months of breastfeeding with infant or maternal ART [5, 6]. Breastfeeding improves child health and development [7–17].

South Africa, a country with a high prevalence of HIV, recently phased out access to free formula milk in the public sector in support of breastfeeding for women living with HIV. Despite the evidence showing reduced risk of infants dying from infections when breastfed, especially in high HIV prevalence settings, breastfeeding rates remain low with challenges to promote breastfeeding by HIV-infected women [7–10]. We conducted a prospective cohort study in South Africa, when the Western Cape prevention of mother-to-child transmission program was phasing out access to free formula milk [18]. We found that few HIV-infected women chose to breastfeed; among those who did, many (50%) switched to formula feeding early (approximately four months following delivery) [18]. Developing simple interventions to promote and sustain continued breastfeeding by women is a public health priority.

Mobile phone text messaging (mHealth) is a simple, low-cost intervention that can promote health behavior change [19, 20]. Increasing mobile phone use in Africa stimulated research efforts on how to leverage mobile phones as a communication tool in healthcare. Text messaging improves adherence to medication among HIV-infected, diabetes, and tuberculosis patients [21–23]. Text messaging not only improves adherence to ART, but also reduces viral load and treatment interruptions [23, 24]. These trials suggest that specific characteristics of the text messages such as interactivity, timing, and content influence text messaging efficacy. Interactive weekly text messaging was superior to interactive daily text messaging [25]. One similarity between adherence to ART and infant feeding practice is that both have a strong behavioral modification aspect: HIV-infected people have to take medication to control the disease while breastfeeding women must modify their feeding practices to improve the health outcomes of their infants. These similarities justify applying information about text messaging from ART adherence studies to infant feeding practices.

Current research suggests that combining a number of approaches is more likely to influence behavior change than an individual approach [26, 27]. Home visits by community health workers and motivational interviewing are interventions known to influence behavior change [28, 29]. However, the former requires considerable human resources and may not be feasible in low-resource settings where the number of healthcare workers is constrained. Patient-centered approaches for negotiating behavior change outperform approaches that instruct patients to change behavior through providing advice [30, 31]. Motivational interviewing is a patient-centered approach that is less coercive and explores the patient’s readiness to change behavior and support the person’s commitment to do so in the preferred direction [32, 33]. Motivational interviewing was of benefit across many health problems, including HIV viral load suppression, body weight, and alcohol and tobacco use [34]. Motivational interviewing is more effective when combined with other interventions [35]. Little is known about the effect of mobile phone text messaging added to motivational interviewing on supporting adherence to breastfeeding among HIV-infected women.

This pilot trial protocol was written following the standard protocol items recommendations for interventional trials (SPIRIT) guidelines (see Additional file 1 SPIRIT checklist) [36].

## Methods and design

### Study design

**Fig. 1:**
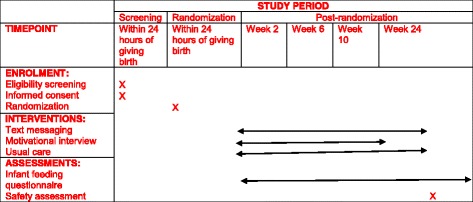
Spirit figure of the pilot trial

We will assess the feasibility of the intervention at a single site: mother–infant pairs will be randomly assigned to interactive mobile phone text messaging plus motivational interviewing or usual standard of care (see Fig. 1).

## Randomization scheme

Participants will be randomly assigned to study groups using a permuted block method. The randomization sequence will be generated using a random number generating program, with a 1:1 allocation ratio with blocks of different sizes to ensure a balanced allocation. Block sizes will be randomly permuted. Electronic sequence generation and random allocation will be done centrally. Women meeting inclusion criteria who consent to participate will be enrolled and immediately assigned a study arm in sequential order. The principal investigator will coordinate the procedures. Only data analysts will be blinded to participant allocation.

## Setting and study population

Women from peri-urban informal settlements will be invited to participate within 24 h of giving birth at a primary healthcare clinic in Cape Town, South Africa.

### Inclusion criteria

HIV-infected mothers will be eligible for inclusion if they initiate breastfeeding within 24 h of giving birth, are on ART, are 18 years or older, own a mobile phone, and their infants are judged to be in good health (ready to be discharged soon after delivery).

### Exclusion criteria

Participants will be excluded if inclusion criteria are not met or if viral load is > 400 copies/mL, if they initiate formula feeding within 24 h of giving birth, gave birth to more than one infant, or if the birth weight is < 2000 g or the gestational age is < 36 weeks. High viral load, multiple birth, and low birth weight are associated with sub-optimal infant feeding practices and poor infant outcomes.

## Study interventions

All women and their infants, irrespective of their study assignments, will receive health services and treatment according to the respective provincial guidelines applicable in the sector during the study. No deviation from existing guidelines will be caused by taking part in this study. Participants will be evaluated soon after giving birth and post delivery until cessation of breastfeeding or until end of the study. Children will be tested for HIV infection at time points already in routine practice (i.e. at birth and ten weeks). We will extract information on the children’s HIV status from the clinical and laboratory records.

### Mobile phone text messaging

Consented new mothers will register their phone numbers into an automated text message (SMS) software (provided by WelTel.org). The first SMS will be sent within the first week following delivery. Every Monday morning, a text message will be sent to women in the intervention group encouraging them to exclusively breastfeed and inquire if they have any problems breastfeeding their infants. Participants will be asked to respond by text within 48 h, indicating that they either do not have a problem or they have a problem and require help. Participants may also use the free ‘call back’ function to request a call from the nurse for complex issues or if cost or literacy is a problem. The research nurse will review all the responses and then follow-up and provide triage to any participants who indicate a problem or call participants who fail to respond within 48 h. To preserve confidentiality, the content of the text messages will not be related to the woman’s HIV status. Participants will be briefly trained on use of the phone and the text messaging protocol.

### Motivational interviewing

In addition to text messaging, women will have individual motivational interviews post delivery at weeks 2, 6, and 10. Study visits will be in line with the Expanded Program of Immunization routine schedule to maximize participation [37]. We will train the research nurse in motivational interviewing techniques, which include reflective listening and expression of acceptance and affirmation. These techniques will enable the research nurse to understand participant’s frame of reference, reinforce participant’s own self-motivational statements, monitor the readiness to change, and affirm the participant’s freedom of choice. Advice will be given with participant’s permission, and when given, the participant will make her own choice. The research nurse will apply these techniques considering the participant’s readiness to change. The interviews will be recorded.

### Usual standard of care

Participants randomized to the usual standard of care group will be counselled by primary healthcare nurses and trained lay counsellors to exclusively breastfeed for the first six months. They will be free to call the clinic staff at any time on their own initiative. Women not receiving motivational interviewing who report adherence concerns during study visits will receive adherence counselling from the primary healthcare nurses and lay counsellors.

All cell phone communications between providers and study participants, in both study arms, will be recorded in a study log.

## Objectives

### Primary objectives

This pilot study will determine the feasibility of conducting a larger trial evaluating the effects of interactive weekly mobile phone text messaging plus motivational interviewing versus usual care in promotion of breastfeeding by HIV-infected women. We will assess the feasibility of the trial for participant recruitment, proportion of eligible participants consenting to participate, and proportion with complete outcome assessment.

### Secondary objectives

To determine if there is a signal of intervention effect on adherence to breastfeeding that may inform larger trial design. We assume text messaging will remind women about the importance of breastfeeding and reinforce regular communication with clinic staff to address adherence related problems. Motivational interviewing may build confidence and motivate women to continue breastfeeding.

## Study endpoints

### Sampling and enrolment

Participants will be enrolled over a period of three months during weekdays.

### Primary endpoints

The primary feasibility outcomes will include the number of participants invited to participate in the study who consent to participate, number of participants with complete evaluation of infant feeding practices at all study visits as assessed by the infant feeding questionnaire, and the number of involuntary disclosures of HIV status due to text messaging. Criteria for feasibility success will include:

- five participants recruited per week (i.e. 60 participants over 12 weeks);
- about 75% of all eligible participants consent to participate;
- complete evaluation of outcomes in at least 70% of all recruited participants;
- breastfeeding adherence rate of at least 70% in the intervention group, at 24 weeks post delivery;
- number of participants reporting involuntary disclosure of HIV status due to text messaging less than 5%.

### Secondary endpoints

Secondary outcomes include number of participants who are exclusively breastfeeding 24 weeks post delivery and number of participants who are breastfeeding. This will be assessed using a questionnaire of food items given to the baby in the last 24 h and one week preceding inquiry. Participants will be considered either “adherent” to exclusive breastfeeding if babies receive only breastmilk and no other liquids or solids based foods or “non-adherent” if other foods are given. Breastfeeding will be assessed by self-report of women who report breastfeeding in addition to giving other foods. Study outcomes will be evaluated at weeks 2, 6, 10, and 24.

## Sample size

We assume that a sample size of 60 participants is large enough to determine feasibility [38]. The information from this pilot trial will inform the design and provide initial estimates of effect for sample size calculation for the main trial.

## Data collection and management

Data collection tools will include a baseline questionnaire and a follow-up questionnaire (at weeks 2, 6, 10, and 24 post randomization). Our questionnaires were developed using a validated WHO infant feeding questionnaire [39]. The questionnaires will be used in English and will be administered by a research nurse or counsellor fluent in the participant’s language. In instances where study participants speak only local languages, the researcher nurse will translate questions directly. We will inquire about feeding practices during the previous 24 h and during the prior week. Study follow-up, SMS messages, and responses will be recorded on a study log weekly. Telephone communications with participants in the usual care group not receiving text messages will be recorded in a similar study log. Clinic records will be reviewed for clinically relevant data. Study follow-up will continue until complete cessation of breastfeeding or until end of study.

To maintain participant confidentiality, only a coded number will identify all questionnaires, reports, and other records. A unique patient identification number will be used to link participant-specific data. All paper records will be kept in a locked file cabinet at the research site and at the Centre for Evidence Based Health Care, Stellenbosch University. Access will be limited to research staff. Electronic files will be password-protected. Clinical information will not be released without written permission of the participant, except if required by the ethics review committee. Participant names will be stored separately. No reports will link individual names with person level data. The research nurse or counsellor will receive training prior to beginning of the trial. A standard operations manual will be available to staff for reference on operational details.

Data will be transcribed from the paper format into an electronic database. We will check data to ensure that entered values are acceptable, required fields are completed, and items are consistent with other related items in the database. We will verify with source documents for any discrepant entries. A record of the database update will be kept, identifying information about the person who made the changes, date, changed values, and comments.

## Analysis plan

### Primary outcomes

Analysis of baseline characteristics and feasibility outcomes will be based on descriptive statistics reported as percentages (95% confidence intervals [CI]) for categorical variables and mean (standard deviation) or median (interquartile range) for continuous variables depending on the distribution.

### Secondary outcomes

We will follow the Consolidated Standards of Reporting Trials (CONSORT) extension for reporting pilot trials [40]. We will use the intention-to-treat principle for secondary outcome analysis, where all participants randomized will be considered per group assignment. In this analysis, we will impute missing outcome data using both the best-case and worst-case scenario. For adherence outcomes, missing data at study visits will be considered as “adherent” and “non-adherent,” respectively. For breastfeeding outcome, missing data at study visits will be considered as “stopped breastfeeding” and “still breastfeeding.” The study is not adequately powered to test intervention effects and these will be of secondary interest to assess potential trends. Logistic regression models will be built for binary outcomes. For timed endpoints, the cumulative proportions of women stopping exclusively breastfeeding and cumulative proportions of those stopping any breastfeeding, we will use the Kaplan–Meier survival analysis followed by multivariable Cox proportional hazards model to adjust for possible baseline imbalances. We will report odds ratios and hazard ratios (95% CI) as initial estimates of effect. Table 1 provides a summary of methods of analysis for each variable. Separately, we will conduct sub-analysis to assess subgroup effects, a summary of this analysis follows below.

**Table 1:** Variables, measures, and methods of analysis

Variable/Outcome	Type	Hypothesis	Outcome measure	Method of analysis
l. Primary				
a. Participants recruited	Count		Percentage recruited	Descriptive statistics
b. Participants consenting	Count		Percentage consenting	Descriptive statistics
c. Completeness of evaluation of outcomes	Count		Percentage with complete outcome evaluation	Descriptive statistics
d. Cumulative breastfeeding adherence at six months	Binary		Percentage adherence in the last 24 h and 1 week	Descriptive statistics
2. Secondary				
a. Adherence to exclusive breastfeeding	Binary	SMS + motivational interview leads to better adherence to breastfeeding than usual care	Percentage adherence in the last 24 h and 1 week	Logistic regression
b. Time to stopping breastfeeding	Time-to-event	SMS + motivational interview prolongs time to stopping breastfeeding than usual care	Reporting of complete cessation of breastfeeding	Kaplan–Meier survival analysis

### Sub-group analyses

Additional analysis of secondary outcomes will include only participants who will be considered as active participants and another analysis of those who will be considered non-active participants. The text messaging intervention requires active participation of study participants to be effective. Participants who respond to the weekly text messages > 80% of the time will be considered as active respondents; those who respond ≤ 80% of the time will be considered as non-active respondents. The 80% cutoff may be revised as appropriate. Participants who never respond to the text message, for whatever reason, will be included in the usual care group. The sample size will be too small to perform further sub-group analysis.

The statistician under the guidance of a senior biostatistician (LT), will conduct all analyses while blinded to study assignment. Stata 14 (StataCorp, College Station, TX, USA) will be used for analysis.

## Ethical aspects

The study will be conducted according to the ethical guidelines and principles of the International Declaration of Helsinki, South African Guidelines for Good Clinical Practice, and the Medical Research Council Ethical Guidelines for Research [41, 42]. Participants will provide written informed consent. Participants will receive R150 (~US $10) for their participation time, transport, lunch on study visit days, and any other study-related costs. There are no anticipated physical risks involved in participating in the study; however, participants will be insured by Stellenbosch University’s research policy in the event of some form of physical injury occurring as a direct result of taking part in this study. The Stellenbosch University Human Research Ethics Committee approved the study protocol (reference no. N16/09/11). This pilot trial will not have a Data and Safety Monitoring Board. The study will not have a codebreaking rule for randomization as the study is unblinded, except for data analysts. All investigators declare no competing interests. If there are any protocol modifications, the ethics committee, trial registry, and trial participants will be informed. Study results will be presented in aggregate format in technical reports and journal publications.

## Discussion

The pilot trial evaluates the feasibility of conducting a larger trial on communication and support approaches that may improve adherence to exclusive and continued breastfeeding by HIV-infected women. Several clinical trials on mobile phone text messaging showed improved treatment adherence for chronic medication, including diabetes, HIV, and tuberculosis. Text messaging and motivational interviewing will allow participants to access personalized adherence advice and support. Text messaging is simple and popular among participants and healthcare providers and this may maximize its use in real practice settings. Additionally, our study is unique in that it tests the ability to combine a number of approaches to influence behavior change.

The study will be conducted in a setting where adherence to breastfeeding is poor. This risks finding larger differences between the intervention and control groups than what could be found in settings with better baseline adherence to breastfeeding. However, we assume the intervention would show a similar trend in direction of effect, in settings with better adherence to breastfeeding. Another disadvantage of our study design is that both healthcare providers and the population whose behavior we are attempting to change (including those in the usual standard of care group) are sensitized to the potential benefits of the intervention during enrolment. We will record the frequency with which participants attend the primary healthcare clinics during follow in order to identify any dose-response relationships between interventions and behavior change.

The design and analysis plan will allow us to assess the effect of the intervention on secondary outcomes. The results of this pilot trial will inform further development of a larger trial on use of mobile phone text messaging plus motivational interviewing to improve breastfeeding practices of HIV-infected women in resource-limited settings. While our measure of behavior change is based on mothers’ self-report rather than more objective measures that accurately predict infant feeding behavior, it is a widely used approach for measuring change in infant feeding practices.

## Trial status

The pilot trial is not yet recruiting participants. Protocol version 1 25/06/2017.

## Additional file

Additional file 1: SPIRIT 2013 Checklist: Recommended items to address in a clinical trial protocol and related documents*. (DOC 118 kb)

## Abbreviations

AIDS: Acquired immunodeficiency syndrome
ART: Antiretroviral treatment
CONSORT: Consolidated Standards of Reporting Trials
HIV: Human immunodeficiency virus
SMS: Short message system
SPIRIT: Standard protocol items recommendation for intervention trials
WHO: World Health Organization
